# Acute Muscle Weakness in Graves' Disease: A Case Report of Hypokalemic Thyrotoxic Periodic Paralysis

**DOI:** 10.7759/cureus.74151

**Published:** 2024-11-21

**Authors:** Wayne A Martini, Thomas N Griffin, Douglas Rappaport, Megan McElhinny, Lauren B Querin

**Affiliations:** 1 Emergency Medicine, Mayo Clinic Arizona, Phoenix, USA; 2 Emergency Medicine, Mayo Clinic Alix School of Medicine, Phoenix, USA

**Keywords:** acute muscle weakness, electrocardiogram abnormalities, goiter, graves' disease, hypokalemic thyrotoxic periodic paralysis (htpp), medication adherence, non-asian population, potassium imbalance, thyroid storm, thyrotoxicosis

## Abstract

Thyrotoxic periodic paralysis (TPP) is a rare but significant complication of hyperthyroidism, characterized by episodes of muscle weakness or paralysis and associated hypokalemia. This case report details a 30-year-old Latin American male with a history of Graves' disease, presenting with acute muscle weakness and hypokalemia. The patient reported transient episodes of weakness over recent weeks, culminating in a severe episode prompting emergency evaluation. Physical examination revealed marked weakness, particularly in the lower limbs, a moderate goiter, and bilateral exophthalmos. Laboratory workup confirmed hypokalemia and uncontrolled thyrotoxicosis, with an elevated Burch-Wartofsky Point Scale score suggestive of thyroid storm.

Treatment involved potassium and magnesium replacement, along with re-initiation of methimazole and propranolol, leading to significant improvement within hours. Persistent thyrotoxicosis was attributed to inconsistent medication adherence, prompting counseling on adherence strategies and discussion of more definitive treatment options. This case highlights the importance of recognizing TPP across diverse populations, emphasizes prompt management of hypokalemia and thyrotoxicosis to prevent severe complications, and underscores the critical role of patient education in chronic disease management. By contributing to the growing body of literature on TPP in non-Asian patients, this report supports the need for heightened clinical awareness of TPP in hyperthyroid patients presenting with acute muscle weakness and hypokalemia. Additionally, it emphasizes the importance of a thorough evaluation, prompt management, and patient education to prevent recurrence and achieve long-term management of Graves' disease.

## Introduction

Thyrotoxic periodic paralysis (TPP) is a rare, but serious complication of hyperthyroidism characterized by muscle weakness or paralysis and hypokalemia. It primarily affects young males of Asian descent. However, cases in non-Asians are being increasingly reported in Western Countries [1,2]. While rare, mortality and significant morbidity associated with TPP have been reported [2]. 

Thyrotoxic periodic paralysis can range from attacks of weakness to mild to complete flaccid paralysis. Proximal muscles usually are more affected than distal muscles. Typically, weakness begins in the lower limbs, progressing to the girdle muscles and then to the upper limbs. Sensory function, along with bladder and bowel function, are typically unaffected [3]. Respiratory muscles are rarely involved, but cases of total paralysis including respiratory and bulbous ocular muscles have been reported [3,4]. 

The pathogenesis of TPP is not fully understood, but it was thought to be related to a sudden intracellular shift in potassium, rather than true potassium deficiency. Several factors have been implicated in this potassium shift, including increased activity of the sodium-potassium pump (Na^+^/K^+^-ATPase) possibly due to direct stimulation by thyroid hormone, or indirectly through increased adrenergic activity, insulin response, and genetic predisposition suggesting associations between TPP and specific human leukocyte antigen (HLA) types as well as single nucleotide polymorphisms (SNPs) near the thyroid hormone response element (TRE) of certain genes [2,3].

## Case presentation

This case highlights the presentation of a 30-year-old Latin American male who arrived at the emergency department (ED) with a chief complaint of weakness getting progressively worse over three days. His medical history includes major depressive disorder, Graves' disease, hypokalemia, and alcohol use disorder, currently in remission. He reported experiencing transient episodes of weakness over the past few weeks, each lasting between 30 to 90 minutes and resolving spontaneously. However, the episode on the day of the presentation did not resolve, which prompted a visit at the ED for more emergent treatment.

Seven months prior, the patient had a similar episode of paralysis with severe hypokalemia, that was theorized to be exacerbated by concurrent use of alcohol. At the current presentation, he reported that he had abstained from alcohol for the last 30 days and that his depression was well-controlled. A review of previous ultrasounds during that admission showed a diffusely heterogeneous and enlarged thyroid gland with increased vascularity, a non-specific sonographic appearance that can be associated with thyrotoxicosis, and no discrete nodules. 

Physical examination revealed significant weakness with an inability to walk. He has a slight increase in weakness on the right side, with 2/5 strength in the upper and lower extremities on the right side and 3/5 strength in the upper and lower extremities on the left side. He had a full passive range of motion of all extremities with intact reflexes. 

**Figure 1:**
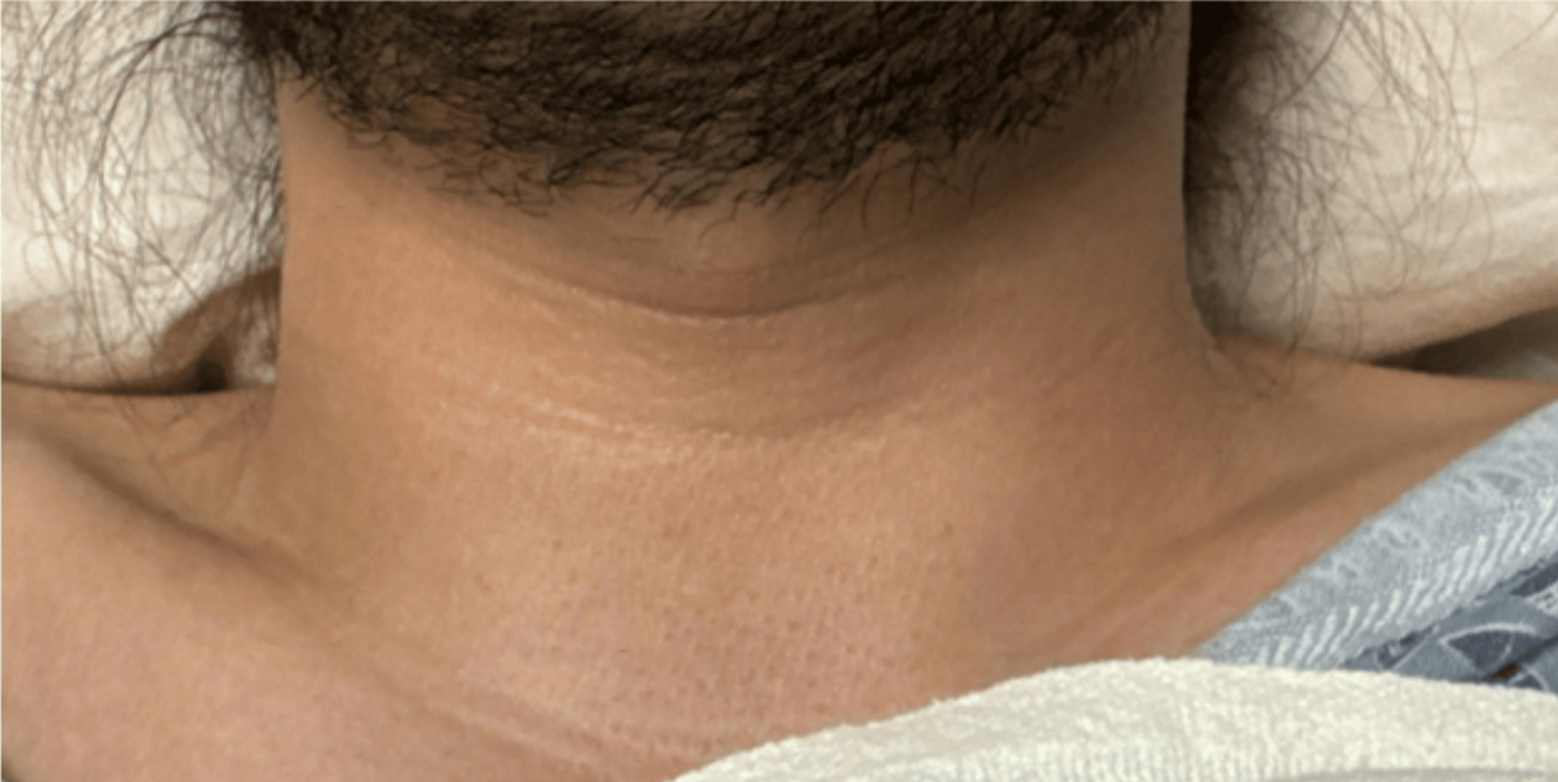
Moderate-sized, diffusely distributed goiter was felt on palpation of the neck without any discernable nodules.

**Figure 2:**
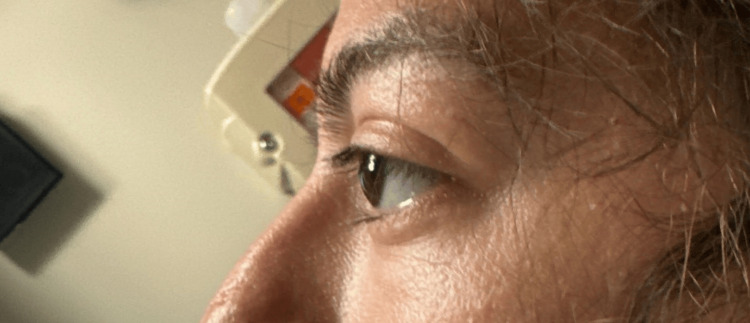
Bilateral exophthalmos with eyes protruding beyond the orbits.

A moderate-sized, diffusely distributed goiter was felt on palpation of the neck without any discernable nodules (Figure 1). An examination of the eyes revealed bilateral exophthalmos with eyes protruding beyond the orbit with lid retraction and delay in the downward movement of the upper eyelid (lid lag). There was a small amount of periorbital edema. No ocular movement restrictions were noted (Figure 2). 

Upon arrival, the patient's vitals were stable, with a blood pressure of 116/86 mmHg, heart rate of 94 beats per minute, temperature of 37.2°C, respiratory rate of 16 breaths per minute, and pulse oximetry reading of 98% saturation on room air. The differential diagnosis for this presentation of acute muscle weakness included Guillain-Barré syndrome, familial hypokalemic periodic paralysis, and primary hyperaldosteronism. Ultimately, the calculation of his Burch-Wartofsky Point Scale (BWPS) for thyrotoxicosis, used to predict thyroid storm, resulted in 50 points, highly suggestive of thyroid storm [4]. This score was due to his slightly elevated temperature, central nervous system depression, nausea with abdominal pain, heart rate in the 90s, and a precipitating stressful event. 

**Figure 3:**
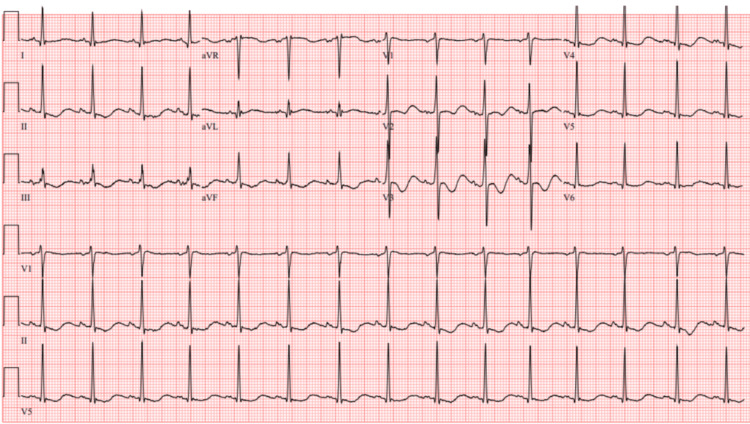
Initial ECG showing T wave inversion, ST depression, and a prominent U wave consistent with hypokalemia.

A point-of-care basic metabolic panel (BMP) revealed low serum potassium levels with an abnormal electrocardiogram (ECG) showing T wave inversion, ST depression, and a prominent U wave, consistent with hypokalemia (Figure 3). Further testing with a formal basic metabolic panel showed low potassium and serum creatinine levels. His thyroid-stimulating hormone (TSH) levels were undetectable and free T4 levels were elevated (Table 1). 

**Table 1:** Point of care and formal basic metabolic panel results.

Parameter	Point-of-Care Result	Formal BMP Result	Reference Range	Interpretation
Potassium (mmol/L)	<2.5	2	3.6 - 5.2	Low
Creatinine (mg/dL)	-	0.52	0.74 - 1.35	Low
Chloride (mmol/L)	-	103	98 - 107	Within Normal Limits
Sodium (mmol/L)	-	138	135 - 145	Within Normal Limits
Thyroid-Stimulating Hormone (TSH, mIU/L)	-	<0.01	0.30 - 4.20	Low (undetectable)
Free T4 (ng/dL)	-	2.5	0.9 - 1.7	High

Prompt resuscitation was started with one liter of intravenous (IV) normal saline with 40 mEq of potassium chloride added at a rate of 250 mL per hour as well as 2 grams of IV magnesium. He was restarted on his home medications of oral methimazole 20 mg twice daily and propranolol 20 mg twice daily. Within several hours, his strength improved noticeably, and by the next morning, he had almost full strength and the ability to walk again. 

**Figure 4:**
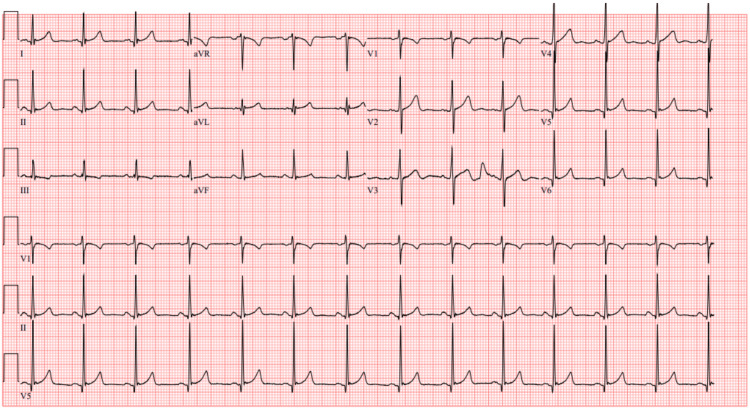
Repeat ECG six hours after arrival showing significant signs of improvement with resolution of his T wave inversions, ST depressions and prominent U wave patterns.

A repeat ECG performed approximately 6 hours after arrival to the emergency department showed significant signs of improvement with resolution of his T wave inversions, ST depressions, and prominent U wave patterns (Figure 4). 

The endocrinology team recommended the continuation of methimazole and propranolol. Despite clinical improvement, his thyroid levels remained high, and medication compliance appeared inconsistent based on patient conversations. The patient was counseled on strategies to improve medication adherence, such as using phone reminders and the iOS health app. The endocrinologist discussed more definitive treatments like surgery or radioactive iodine, but the patient preferred to continue with methimazole and propranolol. 

## Discussion

This case report highlights the complex presentation and management of a 30-year-old Latin American male with a history of Grave’s disease, major depressive disorder, hypokalemia, and alcohol use disorder in remission, who presented with acute muscle weakness. His clinical course underscores the diagnostic challenges and therapeutic considerations in managing hypokalemia in TPP, especially in non-Asian populations.

TPP is a rare but significant complication of thyrotoxicosis characterized by episodes of muscle weakness associated with hypokalemia [5]. While it is more prevalent among Asian populations, this case exemplifies its occurrence in other ethnic groups, including Latin American individuals [5]. The patient's presentation with transient episodes of weakness, coupled with severe hypokalemia and thyrotoxic features, is consistent with TPP. The recurrence of these symptoms, despite a history of hypokalemia-related episodes, suggests an underlying predisposition exacerbated by his thyrotoxic state [2,3]. Patients typically present with symptoms of hyperthyroidism, such as weight loss, palpitations, tremors, and heat intolerance. However, in some cases, hyperthyroid symptoms may be subtle or absent [3].

The patient's physical examination revealed profound muscle weakness, more pronounced on the right side, along with a moderate-sized goiter and bilateral exophthalmos. These findings, along with his biochemical profile of markedly low serum potassium, undetectable TSH, and elevated free T4 were indicative of uncontrolled hyperthyroidism, likely contributing to his TPP episodes. The patient's score on the Burch-Wartofsky Point Scale for Thyrotoxicosis was significantly elevated, indicating a high likelihood of thyroid storm [6]. During paralytic episodes, serum potassium levels are low, but for some patients, total body potassium stores remain normal. This is due to a shift of potassium into cells, and not a loss of potassium from the body [7].

The pathophysiology of TPP involves a rapid shift of potassium from the extracellular to the intracellular compartment, mediated by increased Na^+^/K^+^-ATPase activity [3]. This shift is often triggered by factors such as carbohydrate-rich meals, insulin, or stress. The patient's low serum potassium level (<2.5 mmol/L) and abnormal ECG findings (T wave inversion, ST depression, and prominent U waves) corroborated the diagnosis of hypokalemic paralysis. Prompt management with careful replacement of intravenous potassium and magnesium, along with re-initiation of methimazole and propranolol, resulted in significant clinical improvement within hours, consistent with endocrinology guidelines [3,7]. 

The patient's inconsistent medication adherence, as revealed during further conversation, likely contributed to the recurrence of his symptoms. Thyroid hormone levels remained elevated despite initial treatment due to intermittent medication compliance. The discussion of definitive treatments, such as surgery or radioactive iodine, was offered to the patient; however, he chose to further attempt strict medication adherence with methimazole and propranolol.

## Conclusions

In conclusion, this case report underscores the critical importance of recognizing thyrotoxic periodic paralysis as a rare but significant complication of Graves' disease, even in non-Asian populations where it is less commonly diagnosed. The presentation of severe hypokalemia with acute muscle weakness in the context of hyperthyroidism should prompt clinicians to consider TPP as a differential diagnosis. This case also highlights the valuable role of point-of-care potassium and thyroid testing, coupled with ECG evaluation, in promptly identifying and treating this condition, leading to rapid improvement in patient outcomes. The patient’s clinical course further emphasizes the need for ongoing patient education on the risks associated with medication non-adherence and the benefits of consistent treatment regimens in managing chronic conditions like Graves' disease.

This case serves as a reminder of the complexity of managing TPP in patients with multiple comorbidities, such as depression and a history of alcohol use disorder, which may impact adherence and overall disease control. Through a combination of patient-centered counseling and consideration of definitive therapies like surgery or radioactive iodine, healthcare providers can work collaboratively with patients to achieve sustainable disease management and reduce the risk of recurrent paralysis episodes. Additionally, this case contributes to the growing literature on TPP in diverse populations.
